# Assessment of the hepatoprotective effect of a multi-component preparation containing phospholipids, selected amino acids, betaine, and milk thistle extract in dogs with asymptomatic elevation of liver parameters: a pilot study

**DOI:** 10.3389/fvets.2026.1938591

**Published:** 2026-09-30

**Authors:** Renata Nieradka, Michał Jank, Sebastian Otto, Agnieszka Kurosad

**Affiliations:** 1 Department of Clinical Diagnostics, Faculty of Veterinary Medicine, University of Warmia and Mazury in Olsztyn, Olsztyn, Poland; 2 Department of Pre-clinical Sciences and Infectious Diseases, Faculty of Veterinary Medicine and Animal Sciences, Poznan University of Life Sciences, Poznań, Poland; 3 Vet Planet LLC, Łomianki, Poland

**Keywords:** asymptomatic elevation of liver parameters, dogs, hepatoprotection, nutraceuticals, pilot study

## Abstract

**Background:**

Liver diseases in dogs are often diagnosed at advanced stages due to the organ’s high functional reserve. Early supportive therapy with hepatoprotective nutraceuticals may help slow disease progression in dogs with asymptomatic elevation of liver parameters.

**Aim:**

The aim of this pilot study was to evaluate the hepatoprotective effect of a multi-component preparation containing phospholipids, selected amino acids, betaine, and milk thistle extract in client-owned dogs with asymptomatic elevation of liver parameters.

**Materials and methods:**

Seventeen dogs were included in the open-label study. The animals received Hepatiale Forte Advanced (Vet Expert) for 8 weeks at the manufacturer’s recommended dose. Liver enzyme activities (ALT, AST, ALP, GGT), total bilirubin, serum bile acids, hematological parameters, and abdominal ultrasonography were assessed at baseline and after 8 weeks.

**Results:**

Supplementation resulted in statistically significant reductions in ALT (*p* = 0.004), AST (*p* = 0.004), ALP (*p* = 0.001), total bilirubin (*p* = 0.001) and serum bile acids (*p* = 0.007). A trend toward a decrease was observed for GGT (*p* = 0.052). Hematological parameters remained stable, and the preparation was well tolerated with no adverse effects reported. Ultrasonographic findings were mostly stable or improved (resolution of biliary sludge in some cases).

**Conclusion:**

Eight-week supplementation with the multi-component hepatoprotective preparation was associated with significant improvement in liver biochemical parameters in dogs with asymptomatic elevation of liver parameters. The results support further research in a randomized, placebo-controlled trial.

## 1 Introduction

The liver plays a central role in numerous essential physiological processes, including carbohydrate, lipid, and protein metabolism, detoxification of metabolites and xenobiotics, storage of vitamins, trace elements, fats, and glycogen, as well as immune regulation. Clinical signs and pathological changes associated with liver and biliary tract diseases reflect impairment of these functions. However, due to the liver’s substantial functional reserve (up to 65–70%), overt clinical signs of liver failure, such as jaundice, hypoglycaemia, bleeding tendency, hepatic encephalopathy, and ascites, typically appear only in advanced stages of disease (1, 2). Early clinical signs associated with liver injury or dysfunction, including recurrent inappetence, polydipsia, polyuria, vomiting, and lethargy, are often nonspecific and may also indicate other conditions such as chronic kidney disease, gastrointestinal disorders, endocrine diseases (e.g., diabetes mellitus or hyperadrenocorticism), or systemic infections. Moreover, many liver disorders may remain asymptomatic and are diagnosed incidentally during routine health checks or post-mortem examination (2, 3).

Hepatotoxic factors are commonly classified as inflammatory (viruses, bacteria, rickettsiae, fungi, protozoa, and parasites) or non-inflammatory (metabolic and endocrine disorders, autoimmune processes, toxins, etc.) (2, 4). The pathogenesis of hepatocyte injury and death is multifactorial and not fully elucidated. Key contributing mechanisms include ischemia, hypoxia, oxidative stress from free radicals, deficiency of intracellular substrates, impaired detoxification, and cholestasis. Among these, damage to the hepatocyte cell membrane and subsequent lipid peroxidation are particularly significant, as they can lead to cell disintegration and trigger secondary biochemical and immunological disturbances, ultimately resulting in irreversible intracellular changes and hepatocyte necrosis (1, 2).

Regeneration of the hepatocyte membrane is facilitated by phospholipids, which are rapidly incorporated into both the plasma membrane and the endoplasmic reticulum (4–6). An important aspect of hepatocyte recovery involves suppression of free radical production and enhancement of cellular antioxidant capacity, primarily through increased glutathione levels. This effect can be achieved with antioxidant preparations containing milk thistle extract, vitamins E and C, betaine, and related compounds (7–9). Additionally, effective hepatocyte regeneration requires improved ammonia detoxification, which can be supported by urea cycle amino acids such as arginine or by promoting glutamine synthesis using branched-chain amino acids (BCAAs) (10–12).

The above-mentioned active substances have well-documented hepatoprotective properties in the scientific literature. These effects have been described in humans and, for some components (particularly milk thistle/silybin), also in dogs (13–16). However, the clinical efficacy of their combined use in a single multi-component preparation has not yet been thoroughly described in dogs, particularly in animals with asymptomatic elevation of liver parameter.

## 2 Aim of the study

The aim of this study was to evaluate the hepatoprotective effect of a multi-component preparation containing phospholipids, selected amino acids, betaine and *Silybum marianum* (L.) Gaertn. (milk thistle) extract in client-owned dogs with asymptomatic elevation of liver parameters. This study should be regarded as a pilot investigation.

## 3 Materials and methods

### 3.1 Study inclusion and exclusion criteria

#### 3.1.1 Inclusion criteria

Dogs were eligible for inclusion if they showed elevation of at least one liver enzyme (ALT, AST, ALP or GGT) and/or a bile-related parameter (total bilirubin [TB] or serum bile acids [SBA]) above the laboratory reference range, identified during routine health checks or pre-procedural blood testing, and exhibited no clinical signs of significant hepatopathy (i.e., jaundice, hypoglycaemia, ascites, bleeding tendency or hepatic encephalopathy), nor any other even subtle clinical signs such as vomiting, diarrhea, or reduced or variable appetite.

#### 3.1.2 Exclusion criteria

Dogs were excluded if any of the following were present at baseline: (a) hematological abnormalities suggestive of concurrent systemic disease, including thrombocytopenia (PLT < 100 × 10^9^/L), lymphocytosis exceeding twice the upper reference limit (>10.2 × 10^3^/L), or leukocytosis exceeding twice the upper reference limit (WBC > 33.5 × 10^9^/L); (b) ultrasonographic evidence of severe structural hepatic changes (e.g., diffuse hepatomegaly with a “starry sky” pattern); (c) ultrasonographic evidence of biliary obstruction; (d) concurrent use of medications known to affect liver enzyme activity (e.g., corticosteroids, anticonvulsants, non-steroidal anti-inflammatory drugs, or hepatotoxic antimicrobials); (e) known endocrine disorders (hyperadrenocorticism, diabetes mellitus, or hypothyroidism); or (f) any change in the maintenance diet during the study period.

Asymptomatic ultrasonographic findings, such as mild biliary sludge or gallbladder wall thickening without signs of obstruction, did not constitute exclusion criteria and are reported in the Results section and in Supplementary Table S3.

### 3.2 Experimental protocol

The study was conducted on client-owned dogs presented to the Veterinary Outpatient Clinic of the Faculty of Veterinary Medicine at the University of Warmia and Mazury in Olsztyn, Poland. Twenty dogs were initially screened for enrolment. Dogs were recruited from patients referred for periodic health assessments or pre-procedural blood testing (e.g., prior to dental procedures). After applying the exclusion criteria described above, three dogs (Dogs 1, 3 and 18) were excluded from the final analysis. The group of dogs qualified for the study therefore consisted of 17 animals (*n* = 17). Signalment data (sex, age, breed and body weight) were recorded; detailed individual data are provided in Supplementary Table S4.

Dog 1 was excluded due to severe thrombocytopenia (PLT = 19 × 10^9^/L; reference range 148–484), marked lymphocytosis (11.96 × 10^3^/L), anemia (HGB 11.8 mmol/L, HCT 32.8%) and neutrophilia (13.73 × 10^3^/L), suggesting a probable concurrent systemic disease. Dog 3 was excluded because of critical thrombocytopenia (PLT = 8 × 10^9^/L) and severe lymphocytosis (17.74 × 10^3^/L), raising suspicion of a lymphoproliferative or immune-mediated disorder. Dog 18 was excluded due to marked leukocytosis (WBC 31.76 × 10^9^/L), ultrasonographic evidence of severe diffuse hepatopathy (“starry sky” appearance with hepatomegaly), and a progressive increase in ALP activity during the study (1,654 → 1,870 U/L), consistent with an advanced underlying hepatic or systemic condition rather than an asymptomatic enzyme elevation.

The study lasted 8 weeks. Dogs received Hepatiale Forte Advanced (Vet Expert, Vet Planet LLC) according to the manufacturer’s recommendations: one tablet per 10 kg body weight, once daily. The preparation contained soybean seed extract (*Glycine max* (L.) Merr.), standardized to 98% phospholipids and 20% phosphatidylcholine, L-arginine, L-glutamine, betaine, and milk thistle extract (*Silybum marianum* (L.) Gaertn.), standardized to 60% silybin. The full composition is shown in Table 1. The supplement was administered orally, either directly or mixed with food. Dogs had free access to water, and their usual maintenance diet, which they had been receiving prior to enrolment, was not modified in any way. Owners completed a questionnaire regarding tolerance, palatability and any observed clinical changes (vomiting, diarrhea, appetite, thirst, urination, itching, jaundice, general wellbeing, etc.). No antiparasitic treatments were administered, but topical (spot-on) tick prophylaxis was continued.

**Table 1:** Active substances in the Hepatiale Forte Advanced preparation.

No.	Active substance name	Quantity (in mg per pill)
1	Soybean seed extract (*Glycine max* (L.) Merr.), standardized to 98% phospholipids and 20% phosphatidylcholine	70
2	L-arginine	75
3	L-glutamine	75
4	Betaine anhydrous	70
5	Milk thistle extract (*Silybum marianum* (L.) Gaertn.), standardized to 60% silybin	50

Hematological and biochemical examinations were performed twice: on day 0 and on day 56 (after 8 weeks of the study). Blood samples were collected from the saphenous vein after a 12-h fast into EDTA tubes for hematological analysis (WBC, RBC, HGB, HCT, PLT, neutrophils, lymphocytes, monocytes, basophils and eosinophils) and into serum separator tubes for biochemical analysis (ALT, AST, ALP, GGT, GLUC, ALB, TP, TB and SBA). After clotting at room temperature for 30 min, the samples were centrifuged at 1,500 × g for 10 min to separate the serum. Hematology was analyzed using an ADVIA 2020i (Siemens) analyzer, and biochemistry using an ACCENT-200 (Cormay) analyzer. All analyzes were performed on-site at the clinic’s laboratory. Abdominal ultrasonography of the liver and biliary system was performed at baseline (day 0) and after 8 weeks using the same Arietta 65 (Hitachi) ultrasound machine.

All procedures were performed in accordance with standard veterinary care protocols and relevant Polish legislation (Art. 1, section 2, point 1 of the Act of 15 January 2015 on the Protection of Animals, Journal of Laws 2015, item 266). As the study involved client-owned animals and did not include any experimental interventions beyond routine clinical care, ethics committee approval was not required. Written informed consent was obtained from all dog owners.

### 3.3 Statistical analysis

All statistical analyzes were performed using GraphPad Prism 10 (GraphPad Software, San Diego, CA, USA) and verified with SciPy 1.x (Python 3.x). Normality of paired differences (Day 0 minus Week 8) was assessed using the Shapiro–Wilk test. Because normality was violated for ALT and GGT (*p* < 0.05), the Wilcoxon signed-rank test was used for all paired comparisons. To account for multiple testing of six liver-related parameters, *p*-values were adjusted using the Benjamini–Hochberg procedure (FDR = 0.05). Paired effect sizes were calculated as Cohen’s *d_z_*. Descriptive data are presented as median (IQR), with mean ± SD provided for comparison with previous literature. Adjusted *p*-values <0.05 were considered statistically significant.

## 4 Results

The study group consisted of 17 dogs: 8 females (7 spayed, 1 intact) and 9 males (1 neutered, 8 intact). Body weight ranged from 4.5 to 45 kg (mean ± SD: 19.9 ± 11.7 kg). The age of the dogs ranged from 3 to 13 years (mean ± SD: 7.6 ± 3.2 years). All dogs had a normal body weight; none were overweight or underweight. The study population represented a variety of breeds. Mixed-breed dogs were the most numerous (*n* = 5, 29.4%), followed by German Shepherd Dogs (*n* = 3, 17.6%), Labrador Retrievers (*n* = 2, 11.8%) and Miniature Schnauzers (*n* = 2, 11.8%). Single individuals of the following breeds were also included: Yorkshire Terrier, Beauceron, Chihuahua, short-haired Dachshund and French Bulldog.

Regarding nutrition, the majority of dogs (*n* = 8, 47.1%) were fed exclusively commercial dry food. Five dogs (29.4%) were fed home-prepared diets, three dogs (17.6%) received a mixed diet consisting of both wet and dry food, and one dog (5.9%) was fed exclusively wet food. The diet was not modified in any way throughout the study. Prior to the study, seven dogs were receiving joint-support supplements and one dog was receiving a supplement aimed at improving coat condition. Baseline characteristics of the dogs are summarized in Supplementary Table S4.

According to the owners, the preparation was well accepted, whether given directly or mixed with food. No clinical adverse effects were observed during the study.

### 4.1 Hematological findings

Hematological parameters remained largely stable during the 8-week supplementation period. White blood cell count decreased significantly (*p* = 0.009, Cohen’s *d_z_* = 0.76), as did monocyte count (*p* = 0.049, Cohen’s *d_z_* = 0.51; Table 2). Basophil count increased statistically (*p* = 0.016), but this change was not considered clinically relevant, as all values remained within the reference range. A trend toward reduction was also observed for neutrophils (*p* = 0.062). No clinically relevant changes were noted in erythrocyte parameters, platelet count or other leukocyte subpopulations. All values stayed within or close to the reference ranges. Hematological results of the studied dogs at baseline and after 8 weeks are presented in Supplementary Table S1.

**Table 2:** Hematological parameters at baseline (Day 0) and after 8 weeks of supplementation with Hepatiale Forte Advanced (*n* = 17 dogs).

Parameter	Reference range	Day 0 median (IQR)	Week 8 median (IQR)	Day 0 mean ± SD	Week 8 mean ± SD	Wilcoxon *p*-value	Cohen’s *d_z_*	Statistical significance
WBC (×10^9^/L)	5.05–16.76	10.34 (6.68)	8.12 (3.96)	12.19 ± 5.72	9.61 ± 3.49	0.009	0.76	(*p* < 0.01)^**^
Ery (×10^12^/L)	5.65–8.87	7.06 (0.37)	7.01 (0.28)	7.07 ± 0.60	7.11 ± 0.43	0.887	−0.10	ns
HGB (mmol/L)	13.1–20.5	15.90 (3.00)	16.40 (2.70)	15.82 ± 2.21	16.25 ± 2.01	0.320	−0.33	ns
HCT (%)	37.3–61.7	49.40 (3.40)	50.60 (1.20)	48.82 ± 5.31	49.64 ± 4.92	0.185	−0.36	ns
PLT (×10^9^/L)	148–484	304 (145)	312 (100)	339.1 ± 94.4	335.2 ± 70.5	0.776	0.07	ns
Neut (×10^3^/L)	2.98–11.64	6.30 (4.16)	6.40 (2.01)	7.65 ± 2.69	6.50 ± 1.38	0.062	0.52	ns
Lymph (×10^3^/L)	1.05–5.1	2.14 (1.89)	2.13 (0.56)	2.84 ± 1.54	2.44 ± 0.98	0.190	0.41	ns
Mono (×10^3^/L)	0.16–1.12	0.31 (0.24)	0.20 (0.14)	0.32 ± 0.17	0.25 ± 0.12	0.049	0.51	(*p* < 0.05)^*^
Baso (×10^3^/L)	0.0–0.1	0.02 (0.01)	0.02 (0.01)	0.02 ± 0.03	0.04 ± 0.05	0.016	−0.61	(*p* < 0.05)^*^
Eos (×10^3^/L)	0.06–1.23	0.32 (0.24)	0.27 (0.19)	0.40 ± 0.44	0.34 ± 0.24	0.289	0.18	ns

^*^p < 0.05, ^**^p < 0.01. ns = not statistically significant.

### 4.2 Serum biochemical parameters

Supplementation with Hepatiale Forte Advanced was associated with statistically significant reductions in ALT (*p* = 0.004), AST (*p* = 0.004), ALP (*p* = 0.001), total bilirubin (*p* = 0.001) and serum bile acids (*p* = 0.007). The effect sizes were medium to very large (Cohen’s *d_z_* ranging from 0.65 to 1.11). A trend toward a decrease was observed for GGT (*p* = 0.052; Table 3; Figure 1).

**Table 3:** Serum biochemical parameters at baseline (Day 0) and after 8 weeks of supplementation with Hepatiale Forte Advanced (*n* = 17).

Parameter	Reference range	Day 0 median (IQR)	Week 8 median (IQR)	Day 0 mean ± SD	Week 8 mean ± SD	Wilcoxon *p*-value (raw)	BH-adjusted *p*-value	Cohen’s *d_z_*	Statistical significance
ALT (U/L)	3.0–60.0	171 (331)	61 (140)	307.4 ± 315.7	141.8 ± 183.2	0.0026	**0.004**	0.65	(*p* < 0.01)^**^
AST (U/L)	1.0–45.0	49 (42)	30 (26)	49.8 ± 26.9	31.1 ± 20.6	0.0024	**0.004**	0.94	(p < 0.01)^**^
ALP (U/L)	20.0–155.0	198 (189)	102 (91)	246.0 ± 186.8	138.2 ± 105.2	0.0003	**0.001**	0.86	(*p* < 0.001)^***^
GGT (U/L)	5.0–25.0	17 (14)	15 (7)	20.5 ± 10.5	16.4 ± 7.1	0.0519	0.052	0.52	ns
TB (μmol/L)	<3.4	5.0 (3.4)	3.0 (1.3)	4.5 ± 1.9	2.9 ± 1.0	0.0002	**0.001**	1.11	(p < 0.001)^***^
SBA (μmol/L)	<30	32.1 (37.8)	18.5 (12.5)	29.4 ± 20.7	18.5 ± 11.1	0.0056	**0.007**	0.71	(*p* < 0.01)^**^

^*^p < 0.05, ^**^p < 0.01, ^***^p < 0.001; ns = not statistically significant.

**Figure 1:**
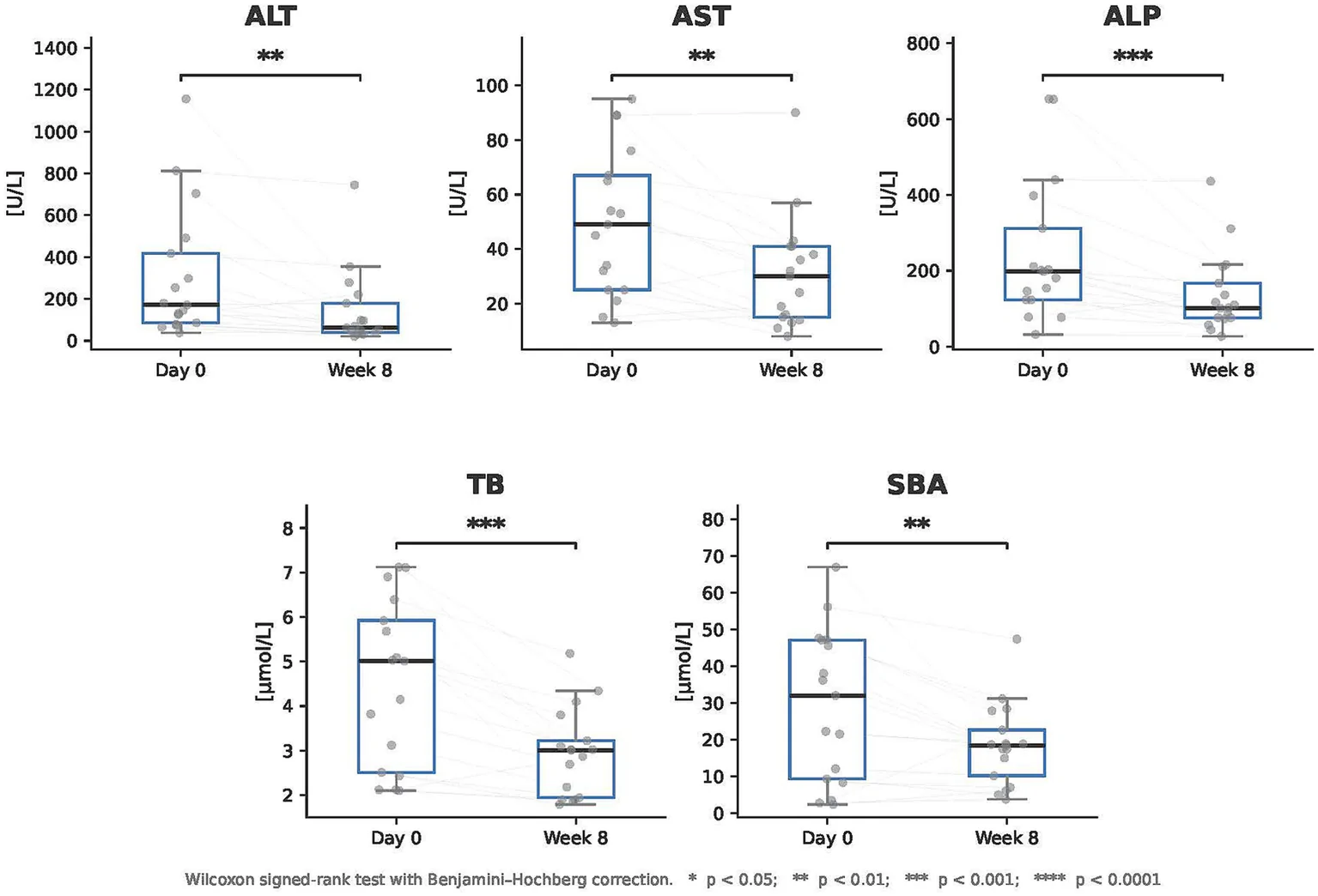
Statistically significant changes in biochemical parameters (ALT, AST, ALP, TB, and SBA) in dogs after 8 weeks of treatment with the Hepatiale Forte Advanced preparation.

No adverse changes in other biochemical parameters (glucose, albumin, total protein) were detected. Biochemical results of the studied dogs at baseline and after 8 weeks are presented in Supplementary Table S2.

### 4.3 Abdominal ultrasonography

Abdominal ultrasonography of the liver and biliary system was performed at baseline (day 0) and after 8 weeks in the 17 dogs included in the final analysis. Evaluation followed standard qualitative criteria used in veterinary practice and included liver size, contour, parenchymal echogenicity and homogeneity, presence of focal lesions, gallbladder wall thickness and content, and bile duct diameter.

At baseline, the liver and biliary system appeared normal in 12 dogs. The hepatic parenchyma showed normal echogenicity and a homogeneous echotexture, with normal liver size and margins. The gallbladder was moderately filled with anechoic bile and had a thin, smooth wall (≤0.1 cm). No cholelithiasis or bile duct dilatation was observed.

Mild abnormalities were detected in five dogs. In dogs 10 and 11, the hepatic parenchyma was hypoechoic with a coarse echotexture, and a small amount of biliary sludge was suspected. In dogs 5, 13 and 20, the liver parenchyma was heterogeneous, with multiple disseminated hyperechoic foci. The gallbladder was enlarged and contained a moderate amount of biliary sludge, with mild thickening of the gallbladder wall and extrahepatic bile ducts, but without ultrasonographic evidence of biliary obstruction.

Follow-up ultrasonography after 8 weeks showed no deterioration. Biliary sludge resolved in dogs 10 and 11. In dog 13, gallbladder filling returned to normal. Diffuse hyperechoic parenchymal lesions persisted in dogs 5 and 20. Dogs 5 and 20 were therefore referred for further diagnostic investigation.

Ultrasonographic findings are summarized in Supplementary Table S3.

## 5 Discussion

Hepatotoxins and various liver-damaging mechanisms commonly induce degradation of membrane phospholipids, leading to impaired membrane integrity and hepatocellular injury. Phosphatidylcholines, the major class of glycerophospholipids, are composed of a glycerol backbone esterified with choline phosphoric acid and long-chain unsaturated fatty acids, predominantly linoleic, linolenic and oleic acid. Owing to their amphiphilic properties and high affinity for damaged membranes, exogenous phosphatidylcholines are readily incorporated into the phospholipid bilayer of hepatocyte cell membranes and the endoplasmic reticulum (4–6). This incorporation supports restoration of membrane fluidity and integrity and may thereby contribute to the maintenance of membrane-dependent processes, including signal transduction, activity of membrane-bound enzymes and transmembrane transport. Furthermore, phosphatidylcholines exert hepatoprotective effects by promoting hepatocyte differentiation and proliferation while inhibiting hepatic fibrogenesis through downregulation of collagen synthesis and upregulation of collagenase activity (17). Betaine (N,N,N-trimethylglycine), serving as a methyl group donor in the methionine cycle, plays a supportive role in the endogenous biosynthesis of phosphatidylcholine via the phosphatidylethanolamine N-methyltransferase (PEMT) pathway (7, 8).

The beneficial effects of phospholipids have been documented in humans and in experimental animals; however, dedicated studies in dogs remain limited.

In humans, polyunsaturated phosphatidylcholine was among the first phospholipids used in patients with chronic active hepatitis and alcoholic liver necrosis, where it was shown to modulate immunological liver injury and support restoration of cell membrane signaling (4). Positive effects on clinical, biochemical, imaging and histological parameters have also been reported in human cases of fatty liver, drug intoxication, viral liver diseases and hepatic coma (4). A hepatoprotective effect has likewise been observed in human patients receiving parenteral nutrition, with a decrease in liver enzyme activity after only 2 weeks of supplementation (18).

In rats, phospholipid supplementation improved clinical condition, laboratory parameters and histopathological changes in alcoholic cirrhosis (19, 36). In carbon tetrachloride-induced liver injury in rats, phosphatidylcholine reduced ALT, AST, ALP, bilirubin and cholesterol, decreased collagen deposition and promoted hepatocyte repair (5, 17, 18, 20). Phospholipids have also been shown in experimental models to improve hepatocyte membrane fluidity, limit apoptosis and stimulate transporters involved in bile secretion (6). In addition, phosphatidylcholine supports intestinal mucosal integrity and may limit bacterial translocation and toxemia associated with liver injury (5).

No specific studies evaluating phospholipid supplementation alone in dogs were identified in this context. These phospholipids are, however, present in the preparation used in the present study (Hepatiale Forte Advanced), which contains soybean seed extract standardized to 98% phospholipids and 20% phosphatidylcholine. Their membrane-stabilizing and hepatoprotective properties may therefore contribute to the reductions in liver enzymes and bile-related parameters observed after 8 weeks of supplementation in the studied dogs.

Liver cell protection also depends on substances that support membrane integrity, redox balance and cellular homeostasis. In Hepatiale Forte Advanced, this role is fulfilled by the combination of L-arginine, L-glutamine, betaine and milk thistle extract.

L-arginine is a conditionally essential amino acid involved in nitric oxide synthesis, urea-cycle activity and protein synthesis (10, 11, 21–23). In humans with liver damage, daily L-arginine administration has been associated with reductions in ALT, AST and ALP (37). In rats, arginine improved hepatic cytoarchitecture, limited oxidative dysregulation and reduced histopathological changes associated with cholestasis or toxic injury (38, 39). *In vitro*, L-arginine reduced hepatocyte injury and chemokine release (24). Overall, its therapeutic effect appears more pronounced than its preventive action (25). These actions may be relevant to the present study, in which supplementation with a preparation containing L-arginine was associated with reductions in ALT and AST in dogs without clinical signs.

L-glutamine is involved in ammonia detoxification and glutathione synthesis (12). In rats, glutamine supplementation supported liver regeneration after partial hepatectomy, increased mitotic activity of hepatocytes and reduced oxidative stress in experimental steatosis (26, 27). In mice, glutamine reduced hepatic iron accumulation and haemolysis (28, 29). Although no dedicated canine studies of glutamine monotherapy were identified, its contribution to antioxidant defense is consistent with the biochemical improvement observed after 8 weeks of supplementation with Hepatiale Forte Advanced.

Betaine acts as a methyl donor and contributes to antioxidant and anti-inflammatory defense (7, 8, 30). In mice, betaine normalized selected methylation-related parameters and reduced experimental hepatic injury (40, 41). In rats, betaine reduced oxidative stress and inflammatory markers and improved hepatocyte structure (31, 32). Human data are less consistent (33, 42). Because triglyceride and cholesterol concentrations were not measured in the present study, no conclusions regarding lipid metabolism can be drawn. The potential relevance of betaine in this study is therefore limited to its antioxidant and cytoprotective actions, which may have contributed to the observed reductions in liver enzymes and bile-related parameters.

Milk thistle extract, a source of silybin and silymarin, has antioxidant, anti-inflammatory and hepatoprotective properties (9, 34). In rats, it reduced toxin-induced liver injury and oxidative stress (9). In dogs, milk thistle or silybin-containing preparations have been associated with reductions in ALT, AST and bilirubin and with changes in selected liver-related markers (13–15, 35). These canine data are particularly relevant, as similar biochemical changes—significant reductions in ALT, AST, ALP, total bilirubin and serum bile acids—were observed in the present 8-week pilot study.

Taken together, the available evidence from humans, rats, mice and dogs supports a hepatoprotective effect of the components of Hepatiale Forte Advanced, based primarily on membrane support and antioxidant activity rather than on a direct improvement in hepatic function.

In the present pilot study, 8-week supplementation with Hepatiale Forte Advanced was associated with statistically significant reductions in ALT, AST, ALP, total bilirubin and serum bile acids. Ultrasonographic examination after 8 weeks showed no deterioration of hepatic or biliary findings, and in some dogs an improvement in hepatic echogenicity or selected biliary parameters was observed. Although ultrasonography was not the primary endpoint of this pilot study, these changes may be consistent with the membrane-stabilizing properties of the preparation. The dogs remained free of clinical signs and the supplement was well tolerated.

Considering that the tested nutrient combination was associated only with favorable hepatoprotective effects over 8 weeks, longer-term use may be considered. Regular blood testing remains essential even in asymptomatic dogs, as early detection and appropriate supportive therapy, including targeted supplementation, may help limit progressive hepatocellular damage and support long-term hepatic homeostasis.

## 6 Limitations

This study has several limitations that should be considered when interpreting its findings. First, the study was designed as an open-label, uncontrolled pilot investigation. The absence of a placebo or untreated control group precludes definitive attribution of the observed improvements to the supplement itself, as spontaneous resolution of enzyme elevations, regression to the mean, or time-dependent changes in hepatic status cannot be excluded as alternative explanations. While the consistency and magnitude of the reductions across multiple independent liver markers argue against a purely stochastic explanation, these findings should be regarded as hypothesis-generating and require confirmation in a randomized, placebo-controlled trial.

Second, the study population, although clinically homogeneous in terms of the absence of clinical signs, was heterogeneous with respect to breed, age, body weight and the potential underlying cause of liver enzyme elevations. Such heterogeneity is inherent to client-owned populations and may have increased variability in treatment response. Some dogs presented with markedly elevated enzymes, while others showed only mild elevations, and the magnitude of the response appeared to vary accordingly. Due to the small sample size (*n* = 17), subgroup or multivariable analyzes of potential confounding factors, including diet, concurrent supplements, age, breed and body weight, were not feasible and would have lacked sufficient statistical power. Future studies should consider stratification by baseline severity or focus on a more narrowly defined population.

Third, the sample size (*n* = 17 after exclusion of three dogs) limits statistical power and the ability to detect smaller effect sizes, particularly for parameters with greater inter-individual variability, such as GGT. The loss of statistical significance for GGT after correction for multiple comparisons may reflect insufficient power rather than a true absence of effect.

Fourth, the underlying cause of liver enzyme elevation was not identified in the majority of cases, and factors known to influence enzyme activity—such as age, dietary composition and individual metabolic variation—were not systematically controlled. Ultrasonographic findings at baseline ranged from normal to diffuse hyperechoic changes and biliary sludge, suggesting a range of underlying hepatic conditions.

Finally, the 8-week follow-up period, while sufficient to demonstrate short-term biochemical improvement, does not allow assessment of long-term outcomes, sustained efficacy, or the durability of the observed effects after discontinuation of supplementation.

## 7 Conclusion

In this open-label pilot study, 8-week supplementation with Hepatiale Forte Advanced was associated with statistically significant reductions in ALT, AST, ALP, total bilirubin and serum bile acid concentrations in dogs with asymptomatic elevations of liver enzymes. The supplement was well tolerated and no adverse effects were observed. While these findings suggest a potential hepatoprotective benefit of the multi-ingredient formulation, the absence of a control group and the small, heterogeneous study population preclude definitive conclusions regarding efficacy. The results are encouraging and support the design of a larger, randomized, placebo-controlled trial to confirm and extend these preliminary observations.

## Data Availability

The original contributions presented in the study are included in the article/Supplementary material, further inquiries can be directed to the corresponding author.
